# Intravitreal injection of aflibercept, an anti-VEGF antagonist, down-regulates plasma von Willebrand factor in patients with age-related macular degeneration

**DOI:** 10.1038/s41598-018-19473-0

**Published:** 2018-01-24

**Authors:** Mariko Yamashita, Masanori Matsumoto, Masaki Hayakawa, Kazuya Sakai, Yoshihiro Fujimura, Nahoko Ogata

**Affiliations:** 1 Department of Ophthalmology, Nara Medical University, Kashihara, Japan; 2 Department of Blood Transfusion Medicine, Nara Medical University, Kashihara, Japan; 3 Japanese Red Cross Kinki Block Blood Center, Ibaraki, Japan

## Abstract

We investigated the association between von Willebrand factor (VWF) and exudative age-related macular degeneration (AMD) in 114 Japanese patients. Intravitreal injection of vascular endothelial growth factor (VEGF) inhibitor is the most effective therapy for AMD. Therefore, we analyzed changes of VWF antigen (VWF:Ag) and VWF multimers (VWFMs) after intravitreal injection of aflibercept, an anti-VEGF antagonist. The relationship between polymorphisms in complement factor H (p.Y402H and p.I62V) and AMD was previously reported. In our patients, p.I62V, but not p.Y402H, was significantly associated with an increased risk of AMD. Pre-treatment plasma levels of VWF:Ag in patients with AMD were significantly higher than those in controls. Unusually large VWFMs (UL-VWFMs) were detected in the majority of AMD patients with concurrent vitreous or subretinal hemorrhage. After intravitreal injection of aflibercept, plasma levels of VWF:Ag and VEGF-A were significantly decreased. UL-VWFMs disappeared after aflibercept injection in three cases, but persisted even 1 month after injection in the other five cases. In conclusion, plasma VWF:Ag levels were significantly elevated in patients with AMD, and decreased after intravitreal aflibercept injection. VWF may play an important role in the pathophysiology of AMD, and aflibercept might improve AMD by reducing plasma levels of VWF in addition to VEGF-A.

## Introduction

Age-related macular degeneration (AMD) is a chorioretinal degenerative disease that occurs mainly in the elderly, and is the leading cause of severe vision loss in industrialized countries^1,2^. Most cases of AMD with vision loss result from pathologic choroidal neovascularization (CNV) in the exudative form of the disease. Vascular endothelial growth factor (VEGF), a key mediator of angiogenesis^3^, was previously shown to be elevated in the eyes of patients with AMD^4^. Intravitreal injection of a VEGF inhibitor is currently the most effective means of arresting CNV in AMD^5,6^. In particular, the intravitreal injection of aflibercept, the latest VEGF antagonist, significantly decreased plasma VEGF concentrations 1 month after injection^7,8^. However, the pathogenesis of AMD is still poorly understood.

Recent association studies determined that the coding variant p.Y402H in the complement factor H (*CFH*) gene was strongly associated with AMD in numerous Caucasian populations^9–11^. The Y402H variant, however, has not shown a significant association with AMD in Asian populations^12,13^. Meanwhile, some studies found that the *CFH* I62V variant was associated with AMD in both Caucasian and Asian populations^14,15^.

von Willebrand factor (VWF) is a multimeric plasma glycoprotein consisting of a single 250-kD subunit, linked by disulfide bonds in a head-to-head and tail-to-tail fashion, that plays an essential role in primary hemostasis through anchoring platelets onto denuded subendothelial matrices^16^. Plasma VWF is synthesized in vascular endothelial cells and released into the circulation as unusually large VWF multimers (UL-VWFMs), the most biologically active form of VWF^17^. In the normal circulation, UL-VWFMs are rapidly degraded under high shear stress into smaller VWFMs by the metalloproteinase ADAMTS13 (a disintegrin and metalloproteinase with thrombospondin type 1 motifs 13)^18^. Deficiency of ADAMTS13 activity (ADAMTS13:AC) induces the accumulation of UL-VWFMs and leads to platelet hyperaggregation under high shear stress, resulting in thrombotic thrombocytopenic purpura (TTP), a life-threatening blood disease^17^.

Notably, recent studies have indicated that CFH reduces VWF multimer sizes through its action as a disulfide-bond reductase or by conferring a heightened susceptibility to ADAMTS13 by binding to VWF^19,20^. Further, Randi and Laffan^21^ more recently reported that VWF controls angiogenesis and vascular maturation through multiple pathways. In 2001, Lips *et al*.^22^ demonstrated increased plasma levels of VWF in patients with exudative AMD, but no further information has been published since then. In this study, therefore, we extensively analyzed plasma VWF and its multimer forms, as well as ADAMTS13, VEGF-A, and *CFH* single nucleotide polymorphisms (SNPs) in Japanese patients with AMD.

## Results

### Characteristics of Study Subjects

One hundred and fourteen patients with exudative AMD, aged between 70.2 and 82.0 years old, and 105 age-matched control subjects with cataracts were enrolled in the study. The characteristics of AMD patients and controls are summarized in Table 1. Exudative AMD was classified into three subtypes, including typical AMD, polypoidal choroidal vasculopathy (PCV), and retinal angiomatous proliferation (RAP). In this study, 70 patients were diagnosed with typical AMD, and the remaining 44 patients were diagnosed with PCV. There were no patients with RAP in this study. Sex and cigarette smoking (past or current) were found to be significantly associated with AMD, consistent with a previous study showing that these were putative risk factors for the disease^23^. However, no significant association was found between AMD and body mass index, dyslipidemia, or hypertension. Further, although it was reported that plasma levels of VWF antigen (VWF:Ag) in individuals with blood group O were lower than in those with non-O blood groups^24^, no significant difference in the distribution of ABO blood groups was found between AMD patients and controls in this study.

**Table 1:** Characteristics of patients with AMD and controls.

	AMD	Control	*p* value
Number	114	105	
Typical AMD, n (%)	70	—	
PCV, n (%)	44	—	
Age, median (IQR), y	77.0 (70.2–82.0)	74.0 (69.0–80.0)	0.10†
Sex (male/female)	82 / 32	59 / 46	0.02‡
BMI, median (IQR), kg/m^2^	22.1 (20.0–23.7)	22.2 (20.3–24.1)	0.56†
Smoking status, n (%)			
Past or current	65 (68.4)	49 (39.5)	<0.001‡
Current	32 (28.1)	23 (21.9)	0.30‡
Blood type, n (%)			
Type O	33 (28.9)	34 (32.4)	0.30‡
Type A	42 (36.8)	38 (36.2)	0.92‡
Type B	21 (18.4)	18 (17.1)	0.80‡
Type AB	18 (15.8)	15 (14.3)	0.80‡
Hypertension, n (%)	54 (47.4)	50 (47.6)	0.97‡
Dyslipidemia, n (%)	9 (7.9)	10 (9.5)	0.70‡

AMD :age-related macular degeneration.

PCV :polypoidal choroidal vasculopathy.

^†^Mann-Whitney U test.

^‡^Chi-squared test.

### Plasma levels of VEGF-A, VWF:Ag, and ADAMTS13:AC

**Figure 1:**
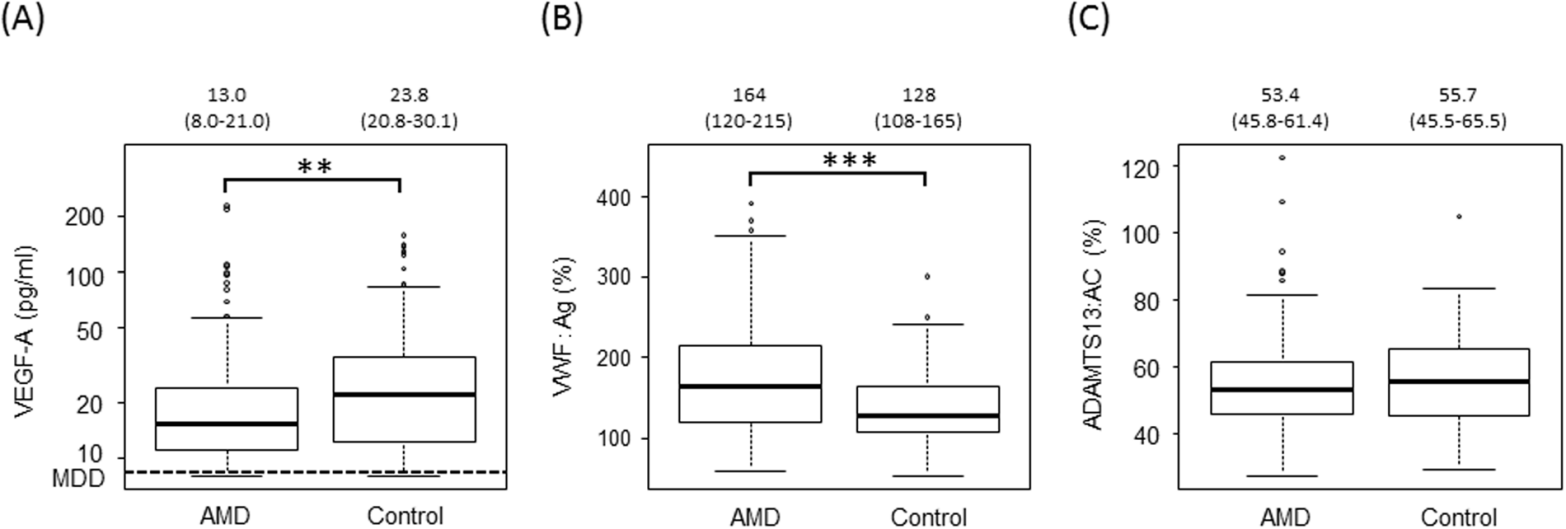
Plasma levels of VEGF-A, VWF antigen, and ADAMTS13 activity in patients with untreated exudative AMD and controls. (**A**) Plasma levels of VEGF-A in patients with AMD were significantly lower than those in controls (**p < 0.01). The minimum detectable dose (MDD) of VEGF-A defined by the manufacturer was 9 pg/ml. (**B**) Plasma levels of VWF antigen (:Ag) in patients with age-related macular degeneration (AMD) were significantly higher than those in controls (***p < 0.001). (**C**) Plasma ADAMTS13 activity (:AC) levels in patients with AMD were almost the same as those in controls.

In contrast to previous reports^7,22^, plasma levels of VEGF-A in untreated patients with AMD were significantly lower than those in controls (*p* = 0.01) (Fig. 1A). The median age of patients in both the AMD and control groups was over 70 years, and plasma levels of VWF:Ag in both groups were higher than those in healthy individuals (aged between 20 and 40 years old). Plasma levels of VWF:Ag in untreated patients with AMD were significantly higher than those in controls (*p* < 0.001) (Fig. 1B). However, plasma levels of ADAMTS13:AC were almost the same in AMD patients and controls (Fig. 1C) and were low compared with those in young, healthy individuals (53.4% and 55.7% vs 100%, respectively).

### VWF multimer analysis in untreated AMD patients

**Figure 2:**
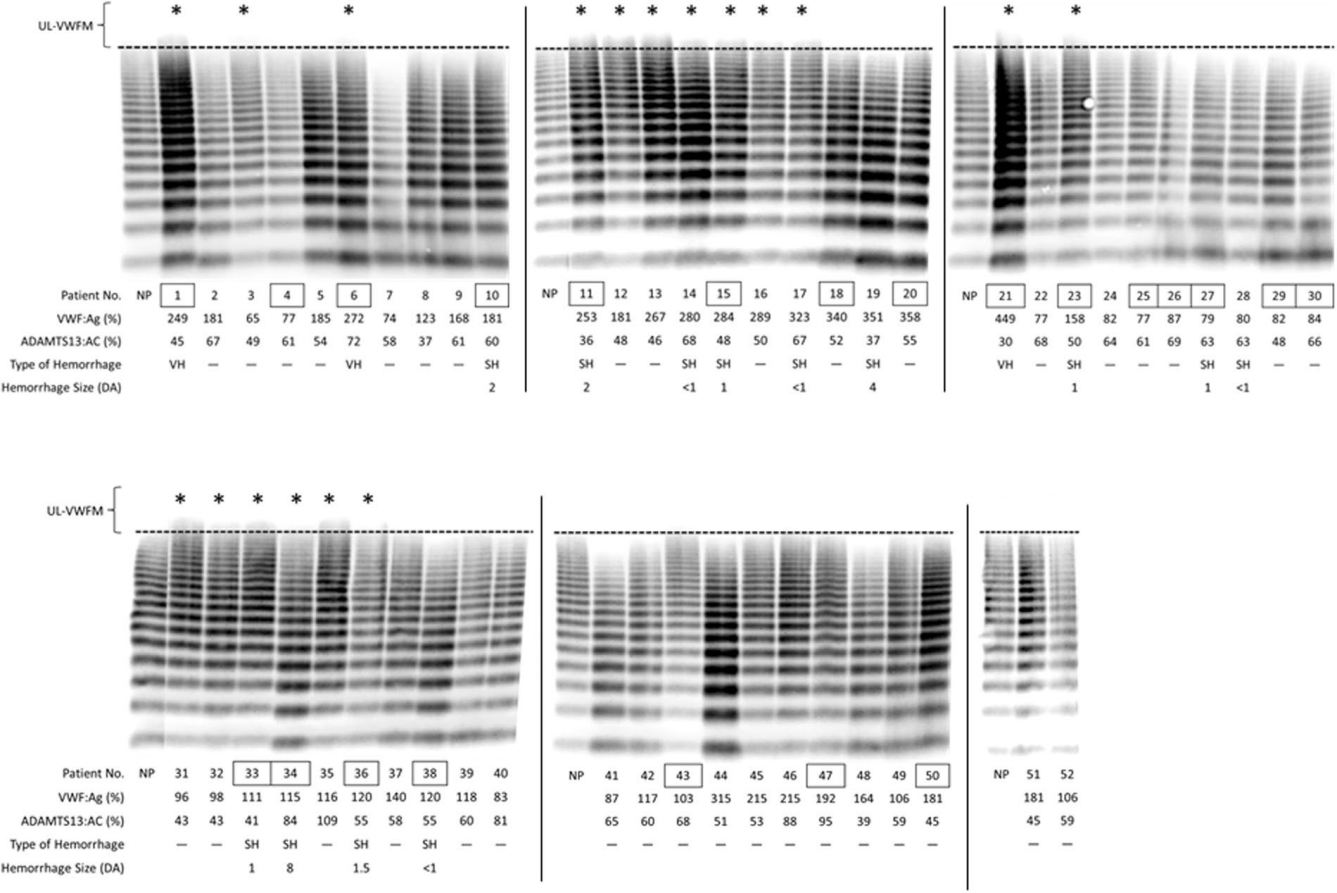
VWF multimer analysis in patients with untreated exudative AMD. Fifty-two out of 114 patients with untreated exudative AMD, patient number (Nos.) 1–52, were shown in this figure. Each patient number enclosed by a square indicates a patient with polypoidal choroidal vasculopathy (PCV). UL-VWFMs were found in 18 patients with AMD, including 9 patients with PCV and 9 with typical AMD. Of these, 3 patients (Nos. 1, 6, and 21) had vitreous hemorrhage (VH) and 6 (Nos. 11, 15, 23, 33, 34, and 36) had subretinal hemorrhage (SH) ≥1 disc area (DA). Asterisks indicate UL-VWFM positivity. NP: normal pool plasma.

**Figure 3:**
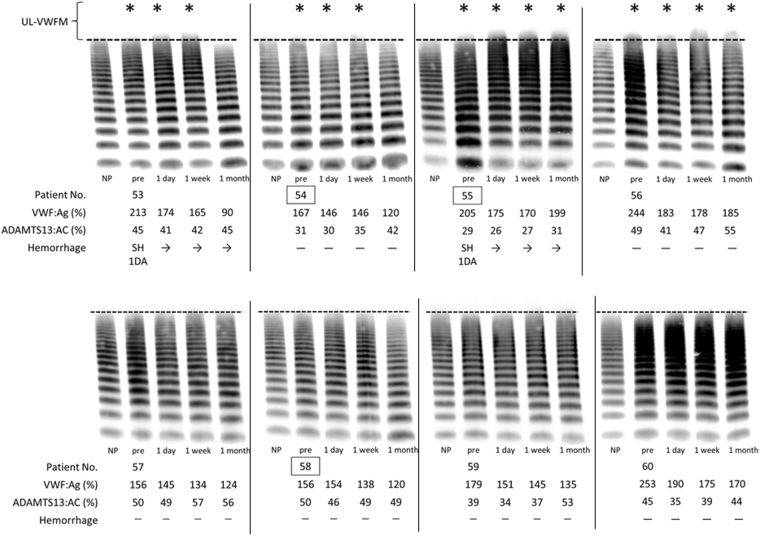
VWF multimer analysis before and after intravitreal injection of aflibercept. We performed VWF multimer analysis in 22 patients with exudative AMD received an intravitreal injection of aflibercept before and at 1 day, 1 week, and 1 month after injection. Of these, VWF multimer analysis of 8 patients (patient Nos. 53–60) were shown in this figure. Each patient number enclosed by a square indicates a patient with polypoidal choroidal vasculopathy (PCV). Patient Nos. 53 and 55 had subretinal hemorrhage and UL-VWFMs before treatment. Patient Nos. 54 and 56 also showed UL-VWFMs before treatment. Patient Nos. 55 and 56 had UL-VWFMs even at 1 month after injection. Asterisks indicate UL-VWFM positivity. NP: normal pool plasma.

As shown in Figs 2 and 3, S1, and S2, VWF multimer analysis was performed in all 114 patients with AMD. Each patient number (No.) enclosed by a square indicates a patient with PCV. Three patients (Nos. 1, 6, and 21) had vitreous hemorrhage (VH), 11 patients (Nos. 10, 11, 15, 19, 23, 27, 33, 34, 36, 53, and 55) had subretinal hemorrhage (SH) ≥ 1 disc area (DA), and 4 patients (Nos. 14, 17, 28, and 38) had SH < 1 DA. Both VH and SH cause severe vision loss.

UL-VWFMs were found in 31 patients (27.2%), including 15 of 44 PCV patients (34.1%) and 16 of 70 typical AMD patients (22.8%). Furthermore, all 3 patients with VH (100%), 8 out of 11 patients with SH ≥ 1 DA (72.4%), 2 out of 4 patients with SH < 1 DA (50.0%) and 18 of 96 patients without ocular hemorrhage (18.7%) had UL-VWFMs. By contrast, UL-VWFMs were not detected in 40 age-matched controls (patients with cataracts) (Supplementary Fig. S3).

### Influence of *CFH* polymorphisms on plasma levels of VWF:Ag, ADAMTS13:AC, and VEGF-A

Previous studies have demonstrated a significant association between the incidence of AMD and *CFH* polymorphisms (p.I62V and p.Y402H)^9–11,14^. As mentioned above, it is now thought that CFH down-regulates VWF multimer sizes via disulfide-bond reductase activity or by increasing VWF susceptibility to ADAMTS13^19,20^.

To further investigate this issue, we first analyzed the SNPs of *CFH* in our patients, and found that the GG genotype in the p.I62V polymorphism was significantly associated with an increased risk of AMD [Odds ratio (OR) = 6.88, 95% confidence interval (CI) = 2.03–23.29], whereas no association was found with the p.Y402H polymorphism (Supplementary Table S1). Further, after adjusting for age, gender, and smoking (past or current), significant associations were still found for p.I62V, but not for p.Y402H (Supplementary Table S1).

Next, we analyzed the relationship between plasma VWF:Ag and *CFH* polymorphisms. There was no association between plasma levels of VWF:Ag and either the p.I62V or p.Y402H mutations (Supplementary Table S2). Further, the *CFH* polymorphisms (p.I62V and p.Y402 H) were not associated with plasma levels of either ADAMTS13:AC or VEGF-A (Supplementary Table S2).

### Changes of plasma levels of VEGF-A, VWF:Ag, and ADAMTS13:AC after the intravitreal injection of aflibercept

**Figure 4:**
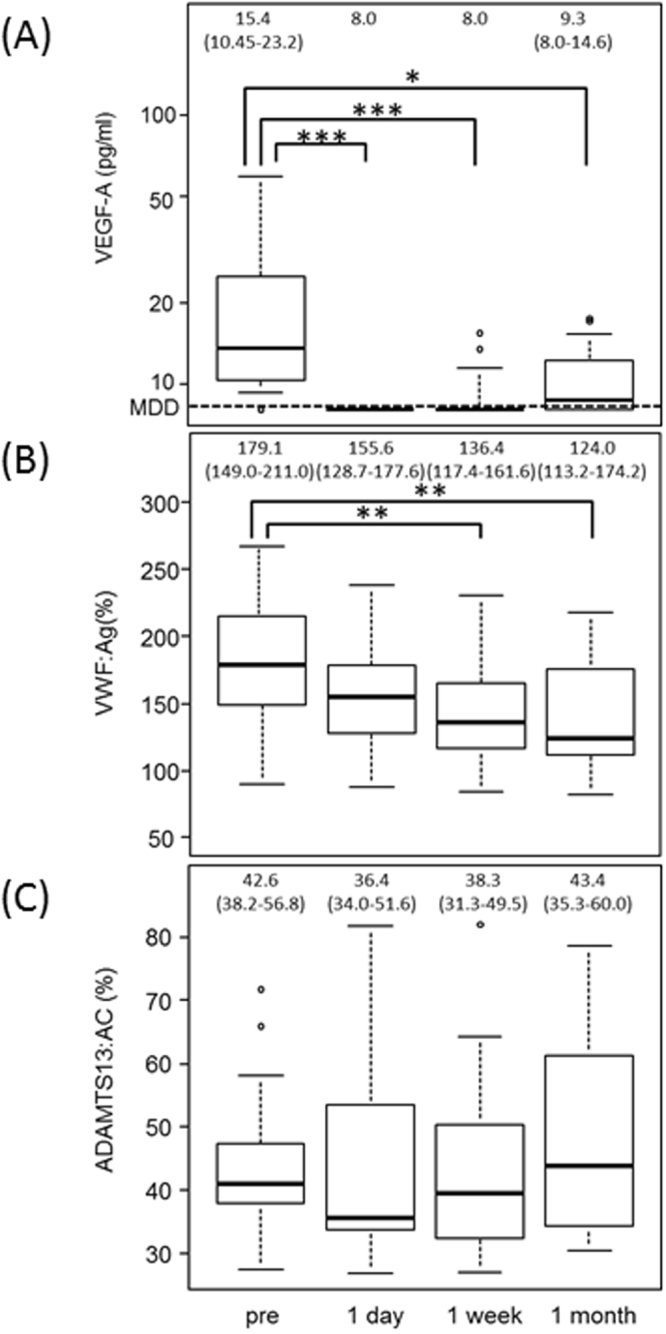
Plasma levels of VWF antigen, ADAMTS13 activity, and VEGF-A before and after intravitreal injection of aflibercept in patients with exudative AMD. (**A**) Plasma levels of VEGF-A were significantly decreased at 1 day after injection (***p < 0.001), and this reduction continued until 1 month after injection (*p < 0.05). (**B**) Plasma VWF:Ag levels gradually decreased after injection, with a significant reduction starting at 1 week (**p < 0.01). (**C**) No significant change was observed in ADAMTS13:AC after injection.

In 82 patients with AMD received intravitreal injections of aflibercept, 22 patients consented to the follow-up analysis after the injection. Plasma levels of VEGF-A, VWF:Ag, and ADAMTS13:AC were analyzed in these 22 patients with AMD at 1 day, 1 week, and 1 month after the intravitreal injection of aflibercept. As shown in Fig. 4A, the mean plasma level of VEGF-A was significantly decreased in all patients at 1 day after the injection (<9.0 pg/ml) compared with before the injection (15.4 pg/ml) (*p* < 0.001). Levels of VEGF-A then gradually recovered, but at 1 week after the injection, 18 of 22 patients still had levels below 9.0 pg/ml (*p* < 0.001). At 1 month after the injection, 11 of 22 patients had levels below the detection limit (*p* = 0.01).

Interestingly, plasma VWF:Ag levels were highly influenced by the intravitreal injection of aflibercept, and thereafter significantly decreased over time (*p* = 0.004 at 1 week after, *p* = 0.003 at 1 month after) (Fig. 4B). However, no significant changes were observed in plasma levels of ADAMTS13:AC during the same period (Fig. 4C).

### Changes of VWF multimers after the intravitreal injection of aflibercept

As shown in Fig. 3 and Supplementary Fig. S2, we investigated VWF multimers in all 22 patients with AMD who were followed up after the intravitreal injection of aflibercept. Eight (Nos. 53, 54, 55, 56, 101, 102, 104, and 109) of the patients had UL-VWFMs before the injection, and 2 (Nos. 53 and 55) of these had current subretinal hemorrhage. In 3 patients (Nos. 53, 54, and 102), UL-VWFMs were disappeared at 1 month after injection. In the other 5 patients (Nos. 55, 56, 101, 104, and 109), UL-VWFMs were still present at 1 month after the injection. One patient (No. 110) had UL-VWFMs at 1 day and 1 week after injection. The remaining 13 patients exhibited no UL-VWFMs at any point during the same 1-month period.

## Discussion

In this study, we showed that pre-treatment plasma levels of VWF:Ag were elevated in patients with AMD compared with controls, and for the first time demonstrated the presence of UL-VWFMs in patients with severe vitreous or subretinal hemorrhage.

VWF is produced in vascular endothelial cells. It is either constitutively secreted or stored in intracellular organelles, termed Weibel-Palade bodies (WPB), from which it is released into the circulation upon vessel injury or in response to various stimuli, including cytokines such as VEGF, epinephrine, and vasopressin^25^. Several studies showed that patients with exudative AMD have severe vascular disturbance^22,26^, which might be related to elevated plasma levels of VWF:Ag as well as enhanced exocytosis of VWF from WPB due to inflammatory cytokines. Thus, accumulation of UL-VWFMs in plasma may induce thrombus formation even in the eye, resulting in vascular injuries and bleeding, both of which may contribute to the development of CNV. However, there is also possibility that both elevated VWF:Ag and the presence of UL-VWFMs in plasma were the consequences of AMD itself.

Several studies have shown that *CFH* p.Y402H is significantly associated with AMD in Caucasian patients^9–11^, but not in Japanese patients^12,27^. This study confirmed the lack of association in a Japanese population, and was also consistent with previous findings showing a correlation between *CFH* p.I62V and Japanese AMD^14,28^.

Nolasco *et al*.^20^ showed that recombinant human CFH functions as a disulfide bond reductase, thereby decreasing the multimeric size of VWF. Hence, we investigated the relationship between *CFH* polymorphisms (p.Y402H and p.I62V) and VWF multimer size, but no clear relationship was found. This, however, might be due to the fact that no patients with *CFH* p.Y402H had the CC allele and only 4 patients with *CFH* p.I62V had the AA allele, indicating that many more patients need to be recruited in future studies before a final conclusion can be reached.

Curiously, in contrast with the findings of Lip *et al*.^22^, pre-treatment plasma levels of VEGF-A in our patients with exudative AMD were significantly lower than those in controls. We are currently unable to explain this discrepancy. Of note, one group also reported that plasma levels of VEGF-A in patients with untreated exudative AMD were lower than those in controls^29^.

In this study, however, intravitreal injection of aflibercept dramatically decreased plasma levels of VEGF-A in AMD patients and also gradually decreased levels of VWF:Ag. The reduction of both VWF:Ag and VEGF-A levels persisted up to 1 month after the injection. Rondaiji *et al*.^25^ reported that VEGF activated the exocytosis of WPBs, which releases VWF from endothelial cells. Aflibercept was shown to have the highest affinity of all VEGF inhibitors to date^30^. Interestingly, local intravitreal injection decreased the systemic levels of both VWF:Ag and VEGF-A in this study. Thus, targeted ocular anti-VEGF therapy appears to reduce plasma levels of VWF:Ag and prevent the development of CNV.

We found that plasma UL-VWFMs disappeared after the intravitreal injection of aflibercept in 3 cases (patient Nos. 53, 54, and 102). However, in the other 5 patients (Nos. 55, 56, 101, 104, and 109) with UL-VWFMs before treatment, these molecules were still present even at 1 month after aflibercept injection. The existence of UL-VWFMs appears to be a potential risk factor for intraocular thrombosis/bleeding or generalized thrombosis in such patients. Fortunately, the abnormal ophthalmological findings in the latter 5 patients improved at 1 month after the injection, but they were still at risk of conditions involving systemic thrombosis or bleeding, such as cerebral infarction or hemorrhage. Notably, such adverse complications of aflibercept have been reported in the literature^31,32^; therefore, further study of this issue is required in conjunction with UL-VWFM analysis.

This study has several limitations. First, it used a cross-sectional design with a relatively small sample size. We will continue this study and evaluate larger numbers of patients to better analyze the pathophysiology of AMD. Second, we sequentially evaluated only patients who were treated with intravitreal injections of aflibercept. It is necessary to compare plasma VWF in patients with and without intravitreal injections to verify the effect of aflibercept. However, it is difficult to analyze AMD patients who have received no prior treatment, because blood tests are not performed in such patients. Finally, we did not evaluate patients for longer than 1 month after the intravitreal injection of aflibercept. Long-term follow-up is necessary to confirm the efficacy of this treatment.

In conclusion, we found an association between plasma levels of VWF:Ag and exudative AMD. Plasma levels of VEGF-A and VWF:Ag were significantly decreased after the intravitreal injection of aflibercept. VWF regulates angiogenesis and could be a new biomarker for AMD. These findings can help expand our knowledge of the pathogenesis of AMD and may be relevant to the potential treatment of this disease.

## Methods

### Subjects

Two hundred and nineteen participants, including 114 treatment-naïve patients with exudative AMD and 105 age-matched controls who were scheduled for cataract surgeries were enrolled. This study was conducted in accordance with the Declaration of Helsinki and was performed after approval from the Institutional Review Board Committee of Nara Medical University. Written informed consent was obtained from all participants.

### Diagnosis of exudative AMD

Exudative AMD was diagnosed primarily based on the ophthalmological findings, spectral-domain optical coherence tomography (SD-OCT), fluorescein angiography (FA), and indocyanine green angiography (ICGA) according to the definition of the Japanese Study Group guidelines^33^. Briefly, exudative AMD was diagnosed in patients who were 50 years or older with abnormalities in the macular area or retinal pigment epithelium (RPE), or with associated neurosensory detachment, retinal hemorrhage, or retinal fibrous scarring without other retinal disorders. Exudative AMD was classified into three types (typical AMD, PCV, and RAP). Eyes with typical neovascular AMD exhibited classic- or occult-type CNV in FA without polypoidal lesions in ICGA and SD-OCT findings of CNV either in the subretinal space or beneath the RPE line. Eyes with PCV exhibited clusters of polypoidal dilation of the vessels with or without abnormal vascular networks in the superficial choroid in ICGA and irregularly elevated RPE line in SD-OCT images. Eyes with RAP exhibited retinal–retinal or retinochoroidal anastomosis in FA or ICGA and retinal swelling with or without RPE detachment in SD-OCT images.

### Injection technique

Eighty-two of 114 patients received intravitreal injections of aflibercept (Eylea; Bayer HealthCare Pharmaceuticals, Berlin, Germany) at a dose of 2.0 mg/0.05 ml. A compounding pharmacy using aseptic methods packaged the medication in a 1.0-ml syringe with an integrated 30-gauge needle. The medication was administered by injection 3.5–4.0 mm posterior to the limbus.

### Blood sampling

Blood samples were collected in tubes containing sodium citrate. Centrifugation was performed at 3,000 g for 15 minutes immediately after sampling, and the obtained plasma was frozen in polypropylene tubes and stored at −80 °C in aliquots until needed for the appropriate assays.

### Genetic analysis

Genomic DNA was extracted from leukocytes using a commercially available genomic DNA extraction and purification kit (QIAmpDNA; Qiagen, Valencia, CA, USA). SNPs of p.Y402H (rs1061170) and p.I62V (rs800292) in *CFH* were genotyped. Polymorphic sites were amplified by polymerase chain reaction (PCR) with specific primers^34^. PCR products were used as the templates for direct DNA sequencing (Applied Biosystems, Foster City, CA, USA) on an automated sequencer (3730xl DNA analyser; Applied Biosystems).

### Assay of VWF antigen (:Ag), ADAMTS13 activity (:AC), and VEGF-A

Plasma VWF:Ag levels were measured by sandwich enzyme-linked immunosorbent assay (ELISA) using rabbit anti–human VWF polyclonal antiserum (DAKO, Glostrup, Denmark)^35^. Plasma levels of ADAMTS13 activity (ADAMTS13:AC) were measured by chromogenic ADAMTS13 activity ELISA (Kinos Laboratories, Tokyo, Japan)^36^. The 100% reference value was defined as the amount of VWF:Ag and ADAMTS13:AC in pooled normal plasma from 20 normal volunteers (10 males and 10 females) aged between 20 and 40 years old. Plasma VEGF-A concentrations were measured by ELISA (Quantikine VEGF ELISA kit; R&D Systems, Minneapolis, MN, USA). The minimum detectable dose (MDD) defined by the manufacturer was 9.0 pg/ml.

### VWF multimer analysis

Multimeric analysis of plasma VWF was performed according to the method of Ruggeri and Zimmerman^37^, with modifications as reported by Warren *et al*.^38^. The experimental conditions, including those for western blotting with luminographic detection, were as previously described by Budde *et al*.^39^. High molecular weight bands that were not detected in normal plasma were defined as UL-VWFMs. The blots were scanned and subjected to densitometric analysis using ImageJ (National Institute of Health, Bethesda, MD, USA). For quantitative analyses, we caluculated the ratio of the densities of UL-VWFMs relative to total VWFM densities. In this study, we judged the positive of UL-VWFM when the ratio of UL-VWFM was over 0.1%.

### Statistical analyses

All statistical analyses were performed using the SPSS 24.0 (IBM Corporation, Armonk, NY, USA) statistical software package. The chi-squared test was used to assess whether the genotype distribution of the controls was in agreement with the Hardy-Weinberg equilibrium. Comparison of genotype frequency between cases and controls was conducted by chi-squared analysis. The logistic regression model was used to analyze the association between AMD and age, gender, smoking status, *CFH* p.Y402H, and *CFH* p.I62V. Odds ratios with 95% confidence intervals were calculated by the chi-squared test to evaluate the association between risk of AMD and *CFH* polymorphisms. Continuous data are given as median and interquartile range (IQR) in parentheses and qualitative data are given as percentages. The Kolmogorov-Smirnov test was used to test for normal distribution. VEGF-A measurements were not normally distributed, and exact values were not given partially because the values were below the minimum detectable dose of 9 pg/ml. Therefore, non-parametric testing was applied and a conservative value of 8 pg/ml was imputed for each value below MDD when performing the calculations. Comparisons between cases and controls were performed using the chi-squared test or Mann-Whitney *U* test as appropriate. Within-treatment-group comparisons were performed with the Friedman and Wilcoxon signed-rank tests. *P* values < 0.05 were considered to be statistically significant.

## Electronic supplementary material

Supplementary dataset
