# Supplementary material for: Intravitreal injection of aflibercept, an anti-VEGF antagonist, down-regulates plasma von Willebrand factor in patients with age-related macular degeneration
Source: Sci Rep. 2018 Jan 24;8:1491. doi: 10.1038/s41598-018-19473-0 (PMC5784081; doi:10.1038/s41598-018-19473-0)
Supplement: Supplementary file 1 — Supplementary dataset [file 41598_2018_19473_MOESM1_ESM.doc]

**Supplemental data**

**Intravitreal injection of aflibercept, an anti-VEGF antagonist, down-regulates plasma von Willebrand factor in patients with age-related macular degeneration**

Mariko Yamashita^1^, Masanori Matsumoto^2^, Masaki Hayakawa^2^, Kazuya Sakai^2^, Yoshihiro Fujimura^3^ & Nahoko Ogata^1^

^1^Department of Ophthalmology, Nara Medical University, Kashihara, Japan. ^2^Department of Blood Transfusion Medicine, Nara Medical University, Kashihara, Japan. ^3^Japanese Red Cross Kinki Block Blood Center, Ibaraki, Japan

Correspondence and requests for materials should be addressed to MM (e-mail: [mmatsumo@naramed-u.ac.jp](mailto:mmatsumo@naramed-u.ac.jp))

**Supplemental Table S1**

**Supplemental Table S2**

**
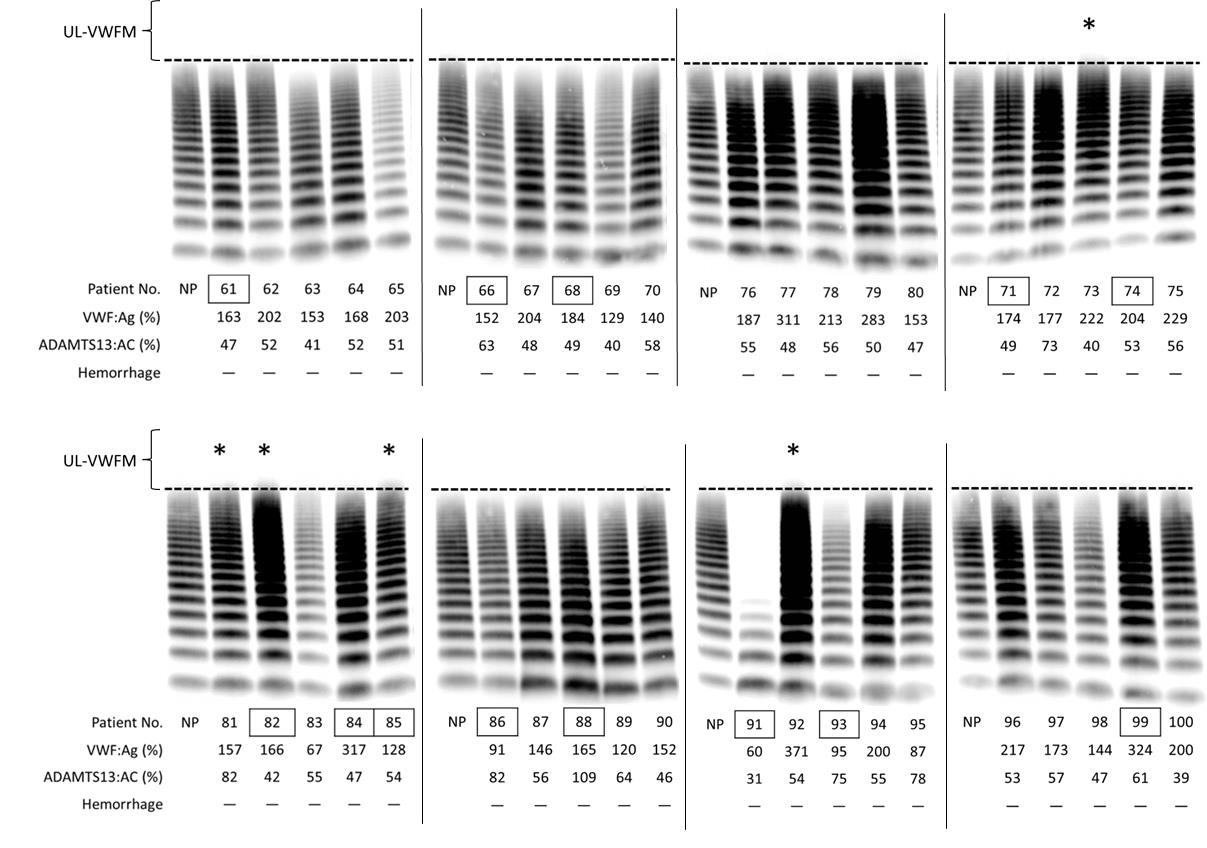
**

**Supplemental Figure S1. VWF multimer analysis in patients with untreated exudative AMD.**

Forty out of 114 patients with untreated exudative AMD, patient number (Nos.) 61-100, were shown in this figure. Each patient number enclosed by a square indicates a patient with polypoidal choroidal vasculopathy (PCV). UL-VWFMs were found in 5 patients　with AMD. Asterisks indicate UL-VWFM positivity. Patient No. 91 without hemorrhage lacked moderate and high molecular weight VWF multimers. NP: normal pool plasma

**
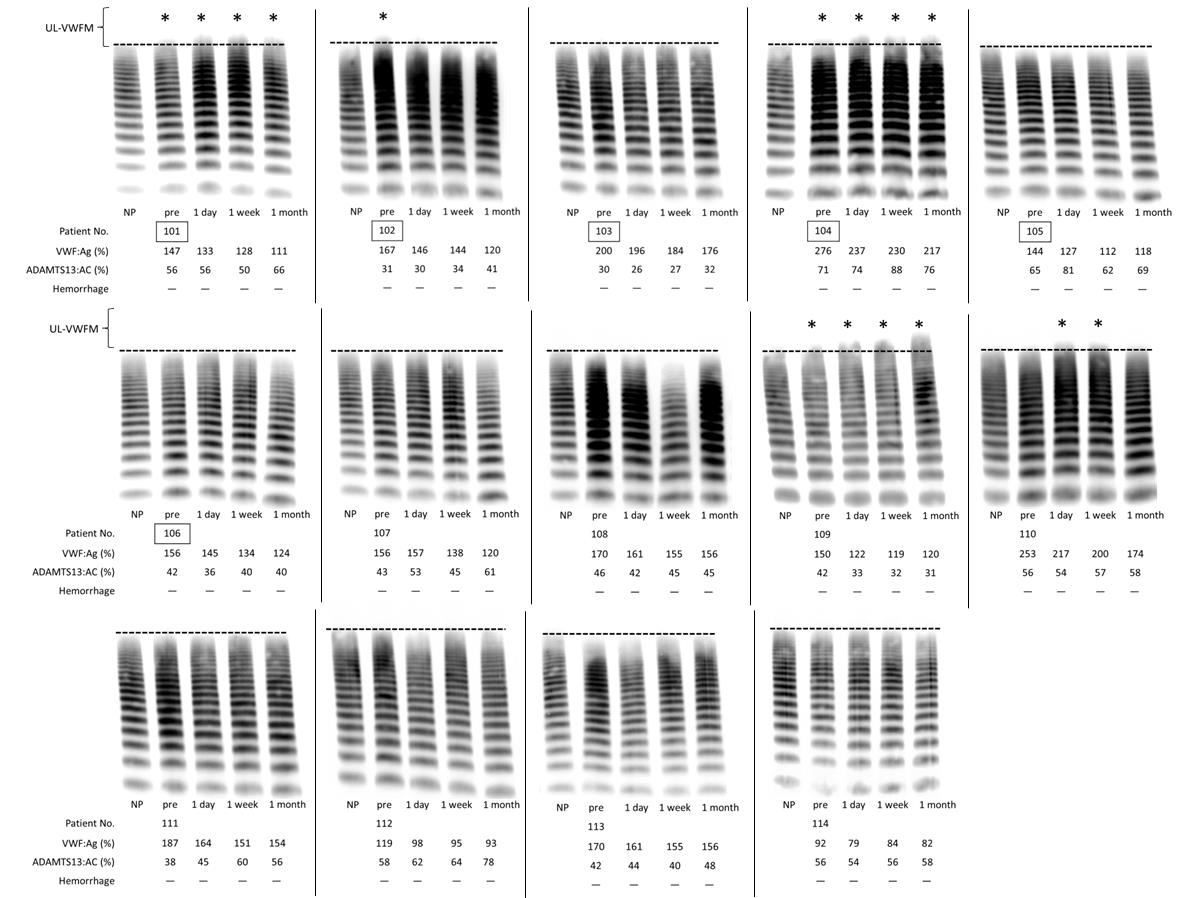
**

**Supplemental Figure S2. VWF multimer analysis before and after intravitreal injection of aflibercept.**

VWF multimer analysis in 14 patints with exudative AMD (patient Nos. 101-114) received an intravitreal injection of aflibercept before and at 1 day, 1 week, and 1 month after injection were shown. Each patient number enclosed by a square indicates a patient with polypoidal choroidal vasculopathy (PCV). Patient Nos. 101, 102, 104, and 109 showed UL-VWFMs before treatment. Asterisks indicate UL-VWFM positivity. NP: normal pool plasma

**
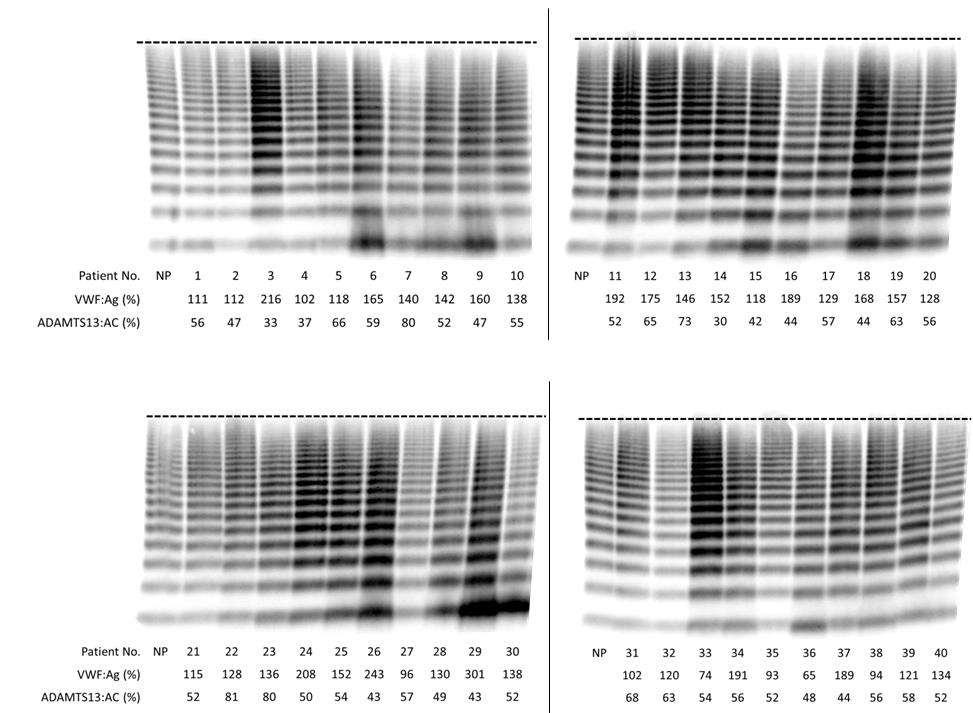
**

**Supplemental Figure S3. VWF multimer analysis in control patients.**

UL-VWFMs were not found in 40 controls scheduled for cataract surgery.
